# HIV virological non-suppression and its associated factors in children on antiretroviral therapy at a major treatment centre in Southern Ghana: a cross-sectional study

**DOI:** 10.1186/s12879-021-06459-z

**Published:** 2021-08-02

**Authors:** Adwoa K. A. Afrane, Bamenla Q. Goka, Lorna Renner, Alfred E. Yawson, Yakubu Alhassan, Seth N. Owiafe, Seth Agyeman, Kwamena W. C. Sagoe, Awewura Kwara

**Affiliations:** 1 Department of Child Health, Korle Bu Teaching Hospital, Accra, Ghana; 2 Department of Child Health, University of Ghana Medical School, Accra, Ghana; 3 Department of Community Health, University of Ghana Medical School, Legon, Accra, Ghana; 4 Department of Health Policy Planning and Management, School of Public Health, University of Ghana, Legon, Accra, Ghana; 5 Department of Immunology, Korle Bu Teaching Hospital, Accra, Ghana; 6 Department of Medical Microbiology, University of Ghana Medical School, Accra, Ghana; 7 Department of Medicine, University of Florida, College of Medicine, Gainesville, Florida USA

**Keywords:** Antiretroviral therapy, Paediatric HIV, Viral load, Virological non-suppression

## Abstract

**Background:**

Children living with human immunodeficiency virus (HIV) infection require lifelong effective antiretroviral therapy (ART). The goal of ART in HIV-infected persons is sustained viral suppression. There is limited information on virological non-suppression or failure and its associated factors in children in resource limited countries, particularly Ghana.

**Methods:**

A cross-sectional study of 250 children aged 8 months to 15 years who had been on ART for at least 6 months attending the Paediatric HIV clinic at Korle Bu Teaching hospital in Ghana was performed. Socio-demographic, clinical, laboratory and ART Adherence related data were collected using questionnaires as well as medical records review. Blood samples were obtained for viral load and CD4^+^ count determination. Viral load levels > 1000 copies/ml on ART was considered virological non-suppression. Logistic regression was used to identify factors associated with virological non-suppression.

**Results:**

The mean (±SD) age of the study participants was 11.4 ± 2.4 years and the proportion of males was 53.2%. Of the 250 study participants, 96 (38.4%) had virological non-suppression. After adjustment for significant variables, the factors associated with non-suppressed viral load were female gender (AOR 2.51 [95% CI 1.04–6.07], *p* = 0.041), having a previous history of treatment of tuberculosis (AOR 4.95 [95% CI 1.58–15.5], *p* = 0.006), severe CD4 immune suppression status at study recruitment (AOR 24.93 [95% CI 4.92–126.31], *p* < 0.001) and being on a nevirapine (NVP) based regimen (AOR 7.93 [95% CI 1.58–1.15], *p* = 0.005).

**Conclusion:**

The prevelance of virological non-suppression was high. Virological non-suppression was associated with a previous history of TB treatment, female gender, severe CD4 immune suppression status at study recruitment and being on a NVP based regimen. Early initiation of ART and phasing out NVP-based regimen might improve viral load suppression in children. In addition, children with a history of TB may need focused measures to maximize virological suppression.

## Background

Human immunodeficiency virus (HIV) infection continues to be one of the most relevant infectious diseases [1, 2]. Antiretroviral therapy (ART) is a critical component of the overall management plan for HIV infection. The primary goal of ART is to suppress viral replication, which ultimately results in restoration of the immune system, reduction in HIV transmission and a general improvement in the quality of life of people infected with HIV [3, 4]. The World Health Organization (WHO) in 2013 recommended HIV viral load (HIV VL) monitoring as the gold standard for monitoring ART effectiveness in resource-limited settings [5]. This recommendation was adopted by Ghana in 2016. According to Ghana’s National AIDS Control Programmme (NACP) guidelines, viral load testing is recommended 6 months after initiating ART and therafter annually for people who have achieved virological suppression [6]. However people with HIV VL levels > 1000 copies/ml are required to undergo intensified adherence support after which the viral load is repeated 3 months later in order to differentiate poor adherence from treatment failure [6].

Virological non-suppression could be due to poor adherence to ART, resistance to ART (transmitted or acquired) or pharmacokinetic issues (poor absorption, under-dosing and drug interactions) [7, 8]. ART regimen-related factors could result in the development of drug resistance [9, 10]. In addition, the delay in introduction of newer, more potent antiretrovirals with high barrier to drug resistance such as second-generation integrase strand transfer inhibitors (INSTIs) in resource-limited settings is a key contributing factor to virolgocial non-suppression.

In previous studies a wide range of factors have been associated with virological non-suppression in children and these include socio-demographic factors such as younger age (less than 3 years) [11], male gender [12, 13], WHO advanced HIV stage [14, 15], co-infection with tuberculosis (TB) [16, 17], nevirapine (NVP) based therapy [18, 19], and poor adherence to treatment [20, 21]. Adherance to ART has always been a challenge for the paediatric population because drug formulations are often less tolerable, and may require dose adjustment as the child grows [22, 23]. Lack of a consistent caregiver in younger children and disclosure issues in adolescents also pose a challenge to medication adherence [22, 23]. These unique issues in children and adolescents can result in virological non-suppression, without the presence of drug resistance mutations [8].

High rates of virological non-supression is common in children in low-and middle-income countries (LMICs) and could be due to poor medication adherence or treatment failure [8]. Identifying patients with virological non-suppression is important for intensified adherence counseling and increased frequency of follow up but it also an important sign of treatment failure especially in persons with good adherence [24]. The aim of this study was to determine the prevalence of virological non-suppression and its associated factors among children living with HIV (CLWH) attending a Paediatric HIV clinic at Teaching Hospital in Ghana. This knowledge would help to target interventions for improving virological suppression, reduce ART failue and ultimately improveclinical care of CLWH.

## Methods

### Study design and setting

This study used a cross-sectional design to recruit paediatric HIV positive patients attending the outpatient clinic from October 2017 to July 2018 at the Korle Bu Teaching Hospital (KBTH), Accra, Ghana. KBTH has 2500 beds and is currently the largest hospital in West Africa and the third largest on the African continent [25]. The hospital is the main tertiary referral centre in Accra and serves the majority of the Southern part of Ghana. The Paediatric HIV clinic at KBTH has been providing comprehensive HIV/AIDS care and management services since 2004. Patients are referred from primary and secondary health facilities as well as from other departments within the hospital. An average of 40 patients are seen per clinic day. The National AIDS Control Program (NACP) provides ART medication free of charge. HIV VL and CD4^+^ counts are also paid for by the program. The clinic uses national treatment guidelines that are in line with current WHO recommendations for ART [26]. Treatment is initiated for all patients irrespective of the CD4^+^ count. Antiretroviral drugs available and in use in various combinations at the clinic at the time of the study were zidovudine (AZT), lamivudine (3TC), abacavir (ABC), efavirenz (EFV), nevirapine (NVP), tenofovir (TDF), and ritonavir boosted lopinavir (LPV/r).

### Study participants

Study participants were CLWH aged between 8 months and 15 years and who had been on ART for at least 6 months. Children who had been transferred from referral points and did not have their previous notes available and children with HIV-2 mono infection were excluded from the study because currently, there are no FDA-approved assays for quantification of HIV-2 RNA. Voluntary informed consent was obtained from parents and guardians of study participants and assent from children aged 10-15 years. Before seeking informed consent/assent, study participants were screened to determine whether they were eligble or not. A questionnaire was administered to study participants and caregivers. Information was also collected retrospectively from their medical records.

### Sample size determination

The Cochran’s sample size formula [27] was used to calculate the sample size to determine the prevalence of non-suppressed viremia. A minimum sample size of 250 study participants was determined using confidence level of 95% and an error margin of 5%. Consecutive cases of HIV children who met the eligibility criteria were enrolled into the study until the sample size was reached.

### Data collection

Participants had their drug doses checked for appropriateness of their current drug regimen. The dosage, (frequency and dose/kg or m^2^) was cross checked with the recommended dosage and appropriateness was documented as ‘yes’ or ‘no.’ ART Adherence was assessed by a pharmacist on the day of recruitment of the study participant using the pill count and this is usually done at every appointment visit.

Pill count (the number of pills taken) was calculated based on the number of unused pills that the care giver brought back when refilling their ART medication on the day of study recruitment [28]. Total refill was the expected number of pills to have been taken since the last visit.

Pill count was callulated as: Total refill - Pill left.

$$ \mathrm{Percentage}\ \mathrm{adherence}\ \mathrm{was}\ \mathrm{calculated}\ \mathrm{as}\;\frac{\mathrm{Pill}\ \mathrm{count}}{\mathrm{Total}\ \mathrm{Refill}}\times 100\% $$

### Laboratory analysis

Laboratory investigations done on the day of recuitment were CD4^+^ count and HIV VL. CD4^+^ absolute cell count and cell percentage were quantified by a dual-platform flow cytometry technology using a FACS Count system (Becton-Dickinson, Franklin Lakes, NJ, USA) according to manufacturer’s instructions at the Fevers Unit Laboratory of KBTH. The HIV RNA VL testing was performed at the Central Laboratory of KBTH using the COBAS® AMPLICOR Monitor test (Roche Diagnostic Systems, Branchburg, NJ, USA), with a a lower limit of detection of 20 copies/ml. The laboratory at the Fevers unit and the Central Lab KBTH are certified by the South African Public Health Reference Laboratory and participates in an external quality assurance testing programme by the South African Public Health Reference Laboratory.

### Operational definitions

In accordance with WHO guidelines, study participants were categorized as having virological non-suppression if the HIV VL level was > 1000 copies/ml on the day of recruitment, after at least 6 months of using ART. Drug Adherence was determined by caregivers report and categorized according to WHO guidelines as follows: Good ≥95%; Fair: 85–94%: Poor < 85% [29].

### Statistical analysis

The dependent variable was virological non-suppression (VL >  1000 copies/ml). The independent variables were the sociodemographic factors, clinical factors and ART Adherence factors. Pearson’s chi-square test of association was used to determine strength of association between the independent categorical variables and the outcome variable (virological non-suppression). The logistic regression model was used in determining the factors influencing HIV VL suppression among study participants with statistical significance set at *p* < 0.05. With the exception of age of the child which was included in the binary logistic regression model because of known associations in the literature [8, 11], all other variables *p*-values < 0.10 from the Pearson’s chi-square test were included. The crude odds ratio (OR) and adjusted odds ratio (AOR) were determined and their respective 95% confidence intervals were calculated.

### Ethical considerations

Ethical apoproval (KBTH-IRB/ 00060/2017) was obtained from the Institutional Review Board of Korle Bu Teaching Hospital, Accra, Ghana. Informed consent was obtained from parents or legal guardians for each minor participant prior to enrolment to participate in the study.

## Results

### Baseline characteristics

The baseline characteristics of the study paticipants are shown in Table 1. Of the 250 participants, 59.2% were within the age range of 10 to 15 years and 53.2% were males. Overall, 46.0% of primary caregivers who were mothers 44% guardians (grandmother, grandfather, aunt, uncle, foster caregiver) and only 10% were fathers. The educational and occupation of parents is described in Table 1. Overall, the mothers of 157 (62.8%) participants were HIV positive while 93 (37.2%) of mothers had unknown HIV status. The unknown status included parents who were dead. The fathers of 62 (24.8%) study participants were HIV positive whilst 105 (42.0%) were HIV negative and 83 (33.2%) had unknown HIV status.

**Table 1:** Baseline characteristics of patients according to virological suppression status

		VL at study recruitment (copies/ml)			
	All patients	Suppressed (≤ 1000)	Non suppressed (> 1000)		
Characteristics	N (%^**1**^)	n (%^**2**^)	n (%^**2**^)	χ^**2**^	***P*** value
**Age category (*N*** **= 250)**				1.1	0.576
< 5 years	30 (12.0)	16 (53.3)	14 (46.7)		
5 to < 10 years	72 (28.8)	44 (61.1)	28 (38.9)		
10 to 15 years	148 (59.2)	94 (63.5)	54 (36.5)		
**Sex of child (*N*** **= 250)**				4.43	0.035*
Female	117 (46.8)	64 (54.7)	53 (45.3)		
Male	133 (53.2)	90 (67.7)	43 (32.3)		
**Primary caregiver relationship to subject (*N*** **= 250)**				2.15	0.499
Mother	115 (46.0)	45 (39.1)	70 (60.9)		
Father	25 (10.0)	12 (48.0)	13 (52.0)		
Guardian	110 (44.0)	50 (45.5)	60 (54.5)		
**Educational status of father (*N*** **= 192)**				4.16	0.527
None	19 (9.9)	11 (57.9)	8 (42.1)		
Basic (Primary/JHS/Middle)	65 (33.9)	36 (40.0)	29(60.0)		
Secondary	82 (42.7)	53 (64.6)	29 (35.4)		
Tertiary	26 (13.5)	19 (73.1)	7 (26.9)		
**Educational status of mother (*N*** **= 168)**				1.27	0.938
None	27 (16.1)	15 (55.6)	12 (44.4)		
Basic (Primary/JHS/Middle)	94 (56.0)	59 (64.0)	35 (36.0)		
Secondary	36 (21.4)	23 (63.9)	13 (36.1)		
Tertiary	11 (6.5)	8 (72.7)	3 (27.3)		
**Occupational class of father (*N*** **= 192)**				Φ	0.541
Professional	2 (1.0)	2 (62.5)	0 (37.5)		
Other	187 (97.4)	115 (61.5)	72 (38.5)		
Unemployed	3 (1.6)	2 (76.5)	1 (23.5)		
**Occupational class of mother (*N*** **= 168)**				Φ	0.73
Professional	0 (0.0)	0 (0.0)	0 (0.0)		
Other	151 (60.4)	90 (59.5)	61 (40.5)		
Unemployed	17 (6.8)	11 (66.7)	6 (33.3)		
**HIV status of father (*N*** **= 250)**				1.83	0.401
Negative	105 (42)	62 (59.0)	43 (41.0)		
Positive	62 (24.8)	36 (58.1)	26 (41.9)		
Unknown	83 (33.2)	56 (67.5)	27 (32.5)		
**HIV status of mother (*N*** **= 250)**				1.97	0.373
Negative	10 (4)	8 (80.0)	2 (20.0)		
Positive	157 (62.8)	98 (62.4)	59 (37.6)		
Unknown	83 (33.2)	48 (57.8)	35 (42.2)		
**Clinical and Lab Variables**					
**TB treatment in the past (*N*** **= 250)**				2.74	0.098
No	179 (71.6)	116 (64.8)	63 (35.2)		
Yes	71 (28.4)	38 (53.5)	33 (46.5)		
**WHO stage at ART initiation (*N*** **= 250)**				1.82	0.611
Stage 1	97 (38.8)	64 (66.0)	33 (34.)		
Stage 2	68 (27.2)	42 (61.8)	26 (38.3)		
Stage 3	63 (25.2)	35 (55.6)	28 (44.4)		
Stage 4	22 (8.8)	13 (59.1)	9 (40.9)		
**Baseline VL (*N*** **= 74)**				Φ	0.751
< 10,000	30 (40.5)	22 (73.3)	8 (26.7)		
10,000 to 99,999	22 (29.7)	13 (60.0)	9 (40.0)		
100,000 to 499,999	12 (16.2)	8 (66.7)	4 (33.3)		
> 499,999	10 (13.5)	7 (70.0)	3 (30.0)		
**Baseline CD4**^+^ **Immune suppression status (overall) (*N*** **= 225)**				1.36	0.71
Severe	35 (15.6)	23 (65.7)	12 (34.3)		
Advanced	25 (11.1)	17 (68.0)	8 (32.0)		
Mild	18 (8.0)	12 (66.7)	6 (33.3)		
none	147 (65.3)	90 (61.2)	57 (38.8)		
**CD4**^+^ **Immune suppression status at study recruitment (overall) (*N*** **= 230)**				Φ	< 0.001*
Severe	24 (10.4)	7 (29.2)	16 (66.7)		
Advanced	18 (7.8)	1 (38.9)	11 (61.1)		
Mild	23 (10.0)	12 (52.1)	11 (47.8)		
normal	165 (71.7)	115 (73.3)	44 (26.7)		
**Art Related Variables**					
**Duration of ART (*N*** **= 250)**				2.15	0.828
< 12 months	32 (12.8)	17 (53.1)	15 (46.9)		
12–23 months	31 (12.4)	19 (61.2)	12 (38.8)		
24–36 months	23 (9.2)	15 (65.2)	8 (34.8)		
36–47 months	20 (8.0)	13 (65.0)	7 (35.0)		
49–50 months	19 (7.6)	10 (52.6)	9 (47.4)		
> 59 months	125 (50.0)	80 (64.0)	45 (36.0)		
**Current ART regimen (*N*** **= 250)**				5.32	0.07
Nevirapine based	68 (27.2)	34 (50)	34(50.0)		
Efavirenz based	173 (69.2)	114 (65.9)	59 (34.1)		
Second line drug	9 (3.6)	6 (66.7)	3 (33.3)		
**Adherence (Pill count percentage rate) (*N*** **= 250)**				7.99	0.018*
Poor adherence (85%)	58 (23.2)	29 (50)	29 (50.0)		
Fair adherence (85–94%)	41 (16.4)	32 (78.0)	9 (22.0.)		
Good adherence (≥ 95%)	151 (60.4)	93 (61.6)	58 (38.4)		
**Person responsible for child’s medication (*N*** **= 250)**				4.55	0.336
Mother only	132 (52.8)	82 (62.1)	50 (37.9)		
Father only	25 (10.0)	13 (52.0)	12 (48.0)		
Both parents	12 (4.8)	5 (41.7)	7 (58.3)		
Grandparents	34 (13.6)	9 (26.5)	25(73.5)		
Others	47 (18.8)	11 (23.4)	36 (76.6)		

χ^2^: Pearson’s chi-square. Φ: Fischer’s exact chi-square test

%^1^: Column percentage. %^2^: row percentage

*: *p* < 0.05

#### Clinical and laboratory details of study participants

Of all participants, 71 (28.4%) had a history of previous TB treatment and 97 (38.8%) had WHO clinical stage 1 disease at initiation of ART.

#### Baseline VL and CD4^+^ count of study participants

Out of the 250 study participants, only 74 (30%) had baseline VL documented. Of the 74 study participants that had records of baseline VL, the median (range) log_10_ copies/ml was 4.8 (1.7–6.9). The breakdown of baseline VL level is shown in Table 1. Of the 74 participants with baseline VL, 30 (40.5%), had baseline VL < 10, 000 copies/ml, and 10 (13.5%) had viral load levels ≥500,000 copies/ml. Overall, of the 225 study participants that had records for their Baseline CD4^+^ counts, 35 (15.6%) had severe CD4 immune suppression status (CD4 < 15% / CD4^+^ count < 200 cells/mm^3^), 25 (11.1%) had advanced CD4 immune suppression status (CD4%: 15–19% / CD4 count: 200–349 cells/mm^3^), 18 (8%) had mild CD4 immune status (CD4%: 20–25% / CD4^+^ count: 350–499 cells/mm^3^) with the majority of study participants 147 (65.3%) having normal CD4^+^ status (CD4 > 25% / CD4 count: > 500 cells/mm) [3].

#### CD4^+^ count at study recruitment

The CD4^+^ results for 20 of the study participants were not available because of technical issues that occurred with their blood samples at the laboratory. Of the 230 that had CD4^+^ results on the day of study recruitment, 10 (4.3%) were children less than 5 years and 220 (95.7%) were children between 5 and 15 years of age. For the overall CD4^+^ immune suppression status, 24 (10.4%) had severe immune suppression status, (CD4 < 15% / CD4 count < 200 cells/mm^3^), 18 (7.8%) had advanced immune suppression status (CD4%: 15–19% / CD4^+^ count: 200–349 cells/mm^3^), 23 (10%) had mild immune suppression status (CD4%: 20–25% / CD4 count: 350–499 cells/mm^3^) and 165 (71.7%) had normal CD4^+^ immune status (CD4%: > 25% / CD4^+^ count: > 500 cells/mm) [3].

#### Proportion of patients with virological non-suppression

At study enrolment, the mean (± SD) duration on ART was 64 months ±3.0 months. Overall, 96 (38.4%) of the 250 participants had virological non-suppression (VL >  1000 copies/ml). Of the 96 participants with virological non-suppression, 20 (20.8%) had VL > 100,000 copies/mL. Overall, 96 (38.4%) of the 250 participant had VL levels < 20 copies/ml and 58 (23.2%) had low-level viraemia (VL levels of 20–1000 copies/ml). For virological non-suppression within the various age groups, 14 (46.7%) were within the age group (< 5 years), 28 (38.9%) were within the age group (5–9 years) and 54 (36.5%) were within the age group (10–15 years).

#### Factors associated with virological non-suppression

Bivariate analysis of factors associated with virological non-suppression are shown in Table 1. Females were more likely to have virological non-suppression in comparison to males (54.2% vs 32.2%, *p* = 0.035). The overall CD4^+^ immune suppression status of the subject at study recruitment showed a significant association with the subjects VL. (Fischer’s exact (Φ, *p* < 0.001). The adherence rate of subject measured by pill count percentage showed a significant association with the subjects VL (χ^2^ = 7.99, *p* = 0.018).

There were no significant differences for the following variables: age of subject, primary caregiver’s relationship to subject, educational status of father, educational status of mother, occupational status of father, occupational status of mother, history of previous TB treatment, WHO stage at initiation of ART, baseline VL and baseline CD4^+^ count values (overall), duration on ART, current ART regimen and person responsible for child’s medication (Table 1).

#### Factors associated with virological non-suppression in multivariate analysis

In multivariate analysis of patients, females were 2.5 times more likely to have virological non-suppression when compared with male study participants (AOR 2.51 [95% CI 1.04–6.07], *p* = 0.041). Additionally, study participants with severe CD4^+^ immune suppression status at study recruitment were 25 times more likely to have virological non-suppression when compared to children with normal CD4^+^ / no immune suppression in multivariate analysis (AOR 24.93 [95% CI 4.92–126.31], *p* < 0.001). Participants with a prior history of TB treatment were 4.95 times more likely to have virological non-suppression as compared to participants without a prior history of TB treatment (AOR 4.95 [95% CI 1.58–15.5], *p* < 0.006). Participants with a NVP based regimen was 7.93 times more likely to be associated with virological non-suppression (AOR 7.93 [95% CI 1.58–15.5], *p* = 0.005). Multivariate analysis of adherence pill count did not yield any significant results (Table 2).

**Table 2:** Univariate and multivariate analysis of factors associated with virological non suppression in CLWH on ART for a at least 6 months (*N* = 250)

	Univariate analysis		Multivariate analysis	
Factor	UOR (95% CI)	***P*** value	AOR (95% CI)	***P*** value
**Sex of child**		0.004*		0.041*
Male	ref			
Female	2.52 1.35–4.67		2.51 1.04–6.07	
**Age category**		0.342		0.138
< 5 years	1.72 0.57–5.24		2.18 0.23–20.89	
5 to < 10 years	1.57 0.78 –3.17		2.84 1.01–8.05	
10 to 15 years	ref		ref	
**CD4**^+^ **Immune suppression status at study recruitment**		< 0.001*		< 0.001*
Severe	9.03 3.08–26.51		24.93 4.92–126.31	
Advanced	7.97 2.03–31.31		9.2 1.27–66.48	
Mild	3.65 1.35–9.9		3.75 0.91–15.4	
None	ref		ref	
**TB treatment in the past**		0.122		0.006*
No	ref		ref	
Yes	1.67 0.87–3.18		4.95 1.58–15.5	
**Current Drug**		0.231		0.005*
Efavirenz based	ref		ref	
Nevirapine based	1.79 0.9 – 3.52		7.93 2.26–27.86	
Second line drug	0.86 0.15–4.86		1.02 0.09–11.8	
**Pill count rate**		0.012*		0.264
Good adherence	ref		ref	
Fair adherence	0.3 0.1–0.93		0.43 0.11 – 1.69	
Poor adherence	1.93 0.92–4.03		1.65 0.54 – 5.01	

*: p < 0.05

## Discussion

In this cross-sectional study, a relatively large proportion of the 250 CLWH (38%) had virological non-suppression after being on ART for a mean period of 64 months. This translates to a virological suppression rate of 62% and is similar to the estimate made by Ghana with respect of its achievement of the third 90 of the UNAIDS 90–90-90 targets. The high rate of non-suppression suggest that intensified efforts to improve HIV treatment in CLWH is needed to achieve the current 95–95-95 targets proposed by UNAIDS with the purporse of ending AIDS by 2030 [30]. Female gender, having a previous history for TB treatment, severe CD4^+^ immunodeficiency status at study recruitment and a NVP-based regimen were associated with virological non-suppression. Some factors identified in other studies such as adherence to ART, clinical stages 3 and 4, parent’s educational level and their employment status were not significant in this current study.

As antiretroviral access continues to expand in resource-limited countries like Ghana, monitoring response to ART by the use of VL measurements is critical in determining the effectiveness of ART in the population [31]. The Sub-Saharan African region’s prevalence for virological non-suppression (> 1000 copies/ml) in children who have been on ART for at least 6 months ranges from 13 to 44% [8, 24, 32]. A virological non-suppression rate of 38% found in this study is concerning given the risk for the emergence of ART resistance and subsequently failure of the ART regimen, necessitating a switch to second, or later third line treatment [8]. This ultimately would result in an increase in morbidity and mortality of the CLWH and cause the spread of resistant viruses [8]. Given these consequences, VL monitoring per national guidelines should be routinely done in all children on ART and those identified as being virologically non-suppressed, should have adherence counselling and then a repeat viral load level to confirm if they have virological failure. Once virological failure has been confirmed then the regimen switch should occur according to Standard Treatment Guidelines outlined by NACP [6].

Rountine VL monitoring is also important for early detection of treatment failure due to pre-treatment drug resistance (PDR), which is known to compromise the efficacy of ART at an individual and population level [33]. Bavara et al using a large database created for surveillance of HIV-1 drug resistance in Italy confirmed that having more than one PDR is an important predictor of virological failure [33]. This phenomenon is on the increase and has also been reported in sub-Saharan Africa [34, 35] Latin America [36] and Asia [37]. While PDR can be detected through baseline genotypic resistance testing (GRT) prior to initiation of ART, it is expensive and not performed at patient entry in care low- and middle-income countries [9]. Currently, VL monitoring has been a programmatic challenge at our Paediatric HIV clinic due to frequent interruptions in the availbility of resources required by the laboratory, resulting in erratic provision of services. Adequate funding and improved logisitics management to ensure uninterrupted VL testing in the laboratory would boost the implementation of the VL monitoring protocol that exisits in the clinic.

The rate of non-suppression children could be due to a number of reasons, depending on the settings. We observed that females were 2.5 times more likely to have virological non- suppression as compared to males. This phenomenon was similar to the study by Muri et al [38], in Tanzanian children, whereby females were also 2.5 more times likely to have virological non-suppression. On the contrary, some studies have reported that males had increased odds of virological non-suppression [13, 39], whilst other studies however found no association between sex and virological non-suppression [40]. The role of gender in virological suppression could be biologic according to authors such as Njom et al. [41]. The relationship of virological suppression and gender is therefore inconclusive and requires further studies.

A third of our study population had been previously treated for TB. We found that having a history of previous TB treatment increased the odds of having virological non-suppression by as much as five times. These findings are in agreement with studies reported by Ahoua et al [42], and by Rajin et al. [43] On the other hand, it has been recently reported that children who had a history of TB co-infection had better virological outcomes [13]. Reasons for this could be due to the close monitoring, frequent clinic visits and adherence support, adopted as part of TB treatment offered at the sample sites. The association of a previous history of TB and virological non-suppression in this current study could be due to the increased pill burden and drug-drug interactions between the medications for TB and HIV therapies, especially the NNRTIs or PIs in the setting of rifampin-containing TB treatment. The significance of TB comorbidity on the occurrence of virological non-suppression buttresses the need for the prioritization of frequent VL monitoring and adherence support in TB/HIV co-infected patients as well as patients who have a history of previous TB. While we were not able to examine the effect of other opportunistic infections (OIs) in this study, it is important to note that non-TB opportunistic infections are now less common than in the past because of early HIV diagnosis and initiation ofeffective ART. As a result, there is reduction of OI-related morbidity and mortality in person with HIV [44].

The odds of virological non-suppression was almost eight times more likely in study participants whose current drug regimen was NVP-based as compared to a study participant who had an EFV-based regimen. The findings of this study is consistent with current literature that shows that patients on NVP-based regimens experience more virological failure than patients on EFV-based regimens [18, 19]. The use of regimens containing NVP is associated with a low genetic barrier of drug resistance and a higher risk of baseline resistance in cases where NVP was used as prophylaxis in the babies for Prevention of Mother To Child Transmission (PMTCT). This current study however did not evaluate prior NVP exposure. Our findings support the current ART guidelines being used at the HIV clinic at KBTH which is to phase out NVP based regimens and replace with LPV/r or EFV regimens for children less than 20 kg. Current guidelines recommend a Dolutegravir (DTG) based regimen as the preferred first-line for children weighing at least 20 kg. Hopefully with the introduction of DTG and its scale up, the non suppression due to certain antiretroviral drugs such as NVP will be reduced.

We observed that study participants found to have severe CD4^+^ immune suppression status at the time of study recruitment were 25 times more likely to have virological non-suppression. These findings are in congruence with studies reported by Jobanputra et al [40], among children in Swaziland and by Izudi et al [45], among children in Northwestern Uganda where it was found that patients with low CD4^+^ count values at study recruitment were more likely to have virological non-suppression. This finding is expected and supports the knowledge that viral suppression leads to immune recovery and could reflect the fact that those study participants who were virologically suppressed had a chance to reconstitute their immune systems for their CD4^+^ counts to increase [46]. This finding also supports early initiation of ART in children and according to the current ART guideline in Ghana, all children confirmed to have HIV diagnosis after birth are started on ART regardless of the CD4+ count.

There was no association between parent-educational level, employment status of parent and virological non-suppression in this study. This is in agreement with a Danish HIV Cohort reported by Legarth et al [47] which also showed no association between education level and virological non-suppression. This finding is however in contrast to a study reported by Mensah *et al* [48] in Ghana, in which a child with an unemployed caregiver was five times more likely to have virological non-suppression. There is substantial evidence on the socioeconomic inequalities in the treatment outcomes of chronic diseases like HIV. This current study could not confirm association between employment status and virological non-suppression. This observation could be due to the fact that for almost a third of study participants, the educational and employment status of parents was not known.

Studies on the relationship between self-reported adherence to ART and virological non- suppression have shown inconsistent results [20, 49]. In this study, adherence level measured by pill count was not associated with virological non-suppression. On the other hand in a clinical trial reported by Intasan *et al* [50], in Cambodian children, non-adherence was associated with virological non-suppression. The measure of adherence used in the study by Intasan *et al* [50], was however the 3 day self -report by caregiver. In a more recent research by Natukunda *et al* [24], in adolescents*,* reported in 2019, more than 70% of adolescents who experienced virological non-suppression were sufficiently adherent as measured by pill count (adherence > 95%). On the other hand, there are also studies whereby poorly adherent patients maintained undetectable VL [49, 51].

Children with low level viraemia (LLV) with detectable viral loads above 20 copies/ml but less than 1000 copies/ml was common (23%) in this study. Thus, these children have the risk of continiuning on a failing regimen for a considerable time, especially given that VL are routinely done once a year. There is therfore the need to design algorithms for patients with LLV to have more frequent VL monitoring as literature has shown the emergence of high-level resistance in this group of individuals .

The strength of this current study is that it did not only determine the prevalence of virological non-suppression but explored factors associated with the phenomenon in children. In the absence of drug resistance testing information, close monitoring of VL levels, multidisciplinary support and prompt clinical judgment are key in ensuring children who have failed treatment are appropriately transitioned to second line therapy. Ultimately, ‘an ounce of prevention is better than a pound of cure.’

### Limitation

The reliance on self-reported data as a measure of adherence, which may be affected by recall and social desirability bias. Analysis of baseline VL and CD4^+^ count was not available for some of the subjects and hence analysis on baseline VL and CD4^+^ could not be done for those patients. This is because VL monitoring was started in April 2011 (as a national policy) and hence all children above 8 years of age did not have the opportunity of having a baseline VL level done.

## Conclusion

The high rate of virological non-suppresion is consistent with findings in other countries in Sub-Saharan setting and emphasizes the great challenge to successfully suppressing HIV in paediatric patients and reinforces the need for regular monitoring of viral load levels in children. Patients on ART with active TB and those with a history of previous TB should be prioritized for more regular VL monitoring (6 monthly) as the national guidelines advocate for yearly monitoring of viral load for patients whose viral load levels are suppressed. Further research should focus on determining resistance patterns in this study population.

## Data Availability

The data sets used and analysed during this study are available with the corresponding author on request.

## Abbreviations

ABC: Abacavir
AIDS: Acquired Immunodeficiency Syndrome
AOR: Adjusted Odds Ratio
ART: Antiretroviral Therapy
CD4: Cluster of differentiation 4
CD4%: CD4 percentage
CLWH: Children Living with HIV
DTG: Dolutegravir
EFV: Efavirenz
ELISA: Enzyme-linked immunosorbent assay
FDA: Food and Drug Administration
HIV: Human Immunodeficiency Virus
HIV VL: HIV viral load
KBTH: Korle Bu Teaching Hospital
LMIC: Low- and middle-income countries
LLV: Low level viraemia
LPV/r: Ritonavir boosted lopinavir
NACP: National AIDS Control Programme
NVP: Nevirapine
PCR: Polymerase Chain Reaction
PDR: Pre-treatment Resistance
PI: Protease Inhibitor
SPSS: Statistical Package for Social Sciences
TEN: Tenofovir
TB: Tuberculosis
VL: HIV-1 RNA Viral Load
U.O.R: Unadjusted Odds Ratio
WHO: World Health Organization
ZDV: Zidovudine
3TC: Lamivudine
